# Ciliary sulcus tube placement may not protect the corneal endothelium in exfoliation glaucoma after Baerveldt glaucoma implantation

**DOI:** 10.1038/s41433-026-04765-x

**Published:** 2026-07-14

**Authors:** Natsumi Murata, Sachi Kojima, Hiro Koshiyama, Yuji Takihara, Eri Takahashi, Toshihiro Inoue

**Affiliations:** Department of Ophthalmology, Faculty of Life Sciences, Kumamoto University, Kumamoto, Japan

**Keywords:** Outcomes research, Risk factors

Exfoliation glaucoma (EXG) has been identified as a risk factor for endothelial cell loss after surgery with glaucoma drainage devices, such as Baerveldt glaucoma implantation (BGI) [1]. Posterior tube placement, such as ciliary sulcus (CS) placement, is generally considered preferable to anterior chamber (AC) placement because it increases the tube–cornea distance and may reduce endothelial damage [2–4]; however, few studies have included patients with EXG, leaving it unclear whether this strategy is equally protective in EXG eyes. Therefore, we examined whether tube placement was associated with 1-year postoperative corneal endothelial cell density (ECD) in pseudophakic EXG eyes after BGI.

This retrospective single-centre study screened 426 consecutive patients who underwent BGI using the BG101-350 (Abbott Medical Optics, Santa Ana, CA, USA) at Kumamoto University Hospital (October 2014–December 2022). Pseudophakic eyes with EXG, no prior glaucoma filtering surgery, and available central ECD data with EM-3000 (Tomey Corp., Nagoya, Japan) at baseline and 1 year postoperatively were included. Eyes with concurrent pars plana vitrectomy, pars plana tube placement, or inadequate specular image quality were excluded. Forty-four eyes from 42 patients met the eligibility criteria and were categorised by tube placement: AC (*n* = 28) or CS (*n* = 16), determined by the surgeon’s discretion based on ocular status. Baseline characteristics were compared using the Mann–Whitney U and Fisher’s exact tests. Analysis of covariance compared 1-year postoperative ECD between groups, adjusting for baseline ECD, age, and preoperative intraocular pressure. A sensitivity analysis excluding eyes with preoperative pseudophacodonesis was also conducted. This study was approved by the Institutional Review Board, and informed consent was obtained using an opt-out approach.

**Fig. 1:**
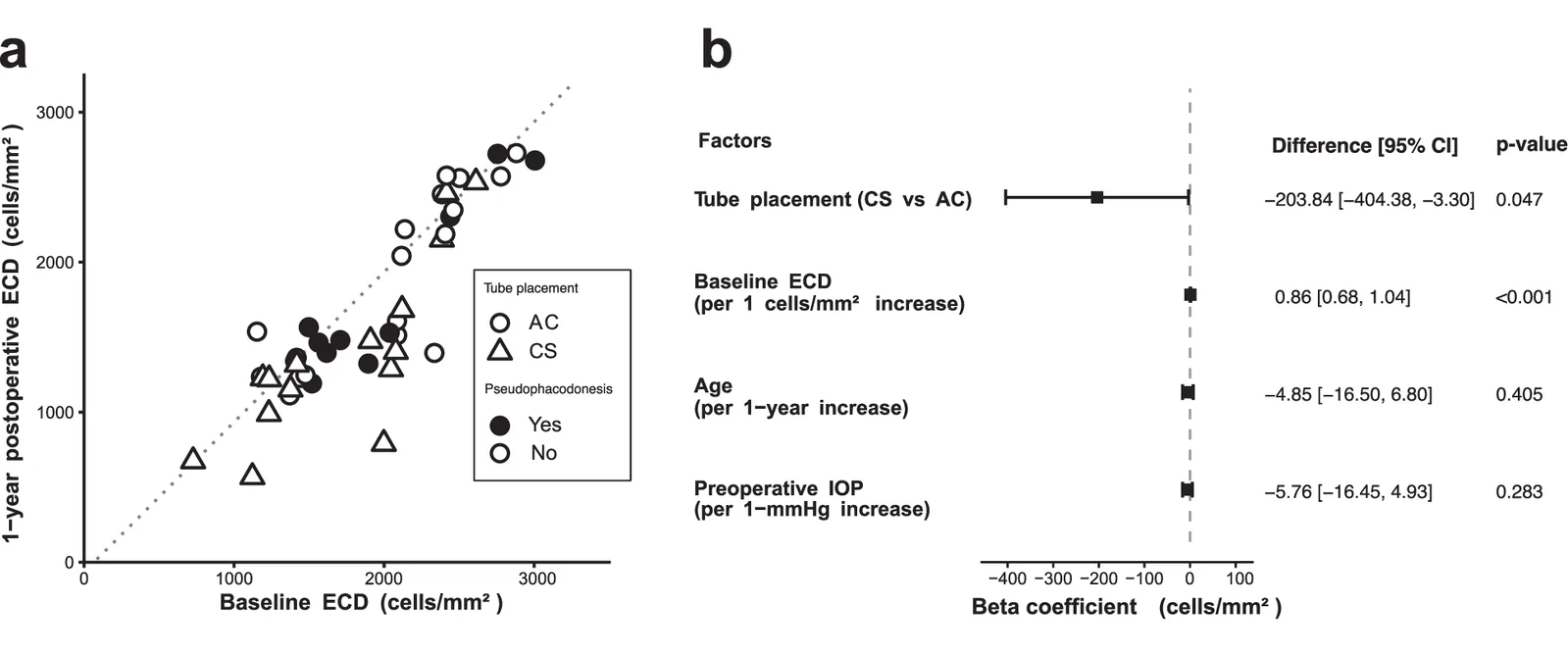
One-year postoperative corneal ECD in eyes with exfoliation glaucoma. **a** Scatter plot of baseline versus 1-year postoperative ECD. Circles indicate AC tube placement, and triangles indicate CS tube placement. Filled symbols indicate eyes with pseudophacodonesis, and open symbols indicate eyes without pseudophacodonesis. The dotted line indicates the identity of the line. **b** Forest plot of the analysis of covariance model for 1-year postoperative ECD. The model included tube placement, baseline ECD, age, and preoperative intraocular pressure. The squares represent the regression coefficients, and the horizontal lines represent the 95% confidence intervals. AC anterior chamber, CS ciliary sulcus, ECD endothelial cell density.

Baseline ECD tended to be lower in the CS group (median 1 664 vs. 2 087 cells/mm^2^; *p* = 0.060), and preoperative pseudophacodonesis was more frequent in the AC group (39% vs. 0%; *p* = 0.003) (Table 1). At 1 year postoperatively, median ECD was significantly lower in the CS group (1257 vs. 1552 cells/mm^2^; *p* = 0.006). Figure 1a shows the distribution of baseline and 1-year postoperative ECD according to the tube location. After covariate adjustment, CS placement was associated with a significantly lower 1-year ECD than AC placement (adjusted difference: −204 cells/mm^2^, 95% CI: -404 to −3; *p* = 0.047; Fig. 1b). The sensitivity analysis excluding cases with pseudophacodonesis was consistent with the primary analysis (*p* = 0.036).

**Table 1:** Baseline characteristics of exfoliation glaucoma eyes stratified by tube placement.

Characteristics	AC (*N* = 28)	CS (*N* = 16)	*p*-value
Age, years			0.271
Median [Q1, Q3]	78 [71.0, 87.0]	76 [70.0, 78.0]	
Mean (SD)	79.0 (9.0)	77.0 (7.0)	
Preoperative IOP, mmHg			0.59
Median [Q1, Q3]	30 [24.0, 35.2]	29 [23.2, 34.0]	
Mean (SD)	30.5 (9.1)	30.7 (9.8)	
Preoperative medication score			0.474
Median [Q1, Q3]	5 (4, 5)	5 (4, 5)	
Mean (SD)	4.3 (0.9)	4.4 (0.9)	
Baseline ECD			0.06
Median [Q1, Q3]	2087 [1509.0, 2429.0]	1664 [1233.0, 2099.0]	
Mean (SD)	2023.0 (542.0)	1705 (556.0)	
Postoperative ECD (1year)			0.006**
Median [Q1, Q3]	1552 [1379.0, 2401.0]	1257 [1071.0, 1579.0]	
Mean (SD)	1794.8 (587.7)	1384.0 (557.0)	
Preoperative pseudophacodonesis	11 (39%)	0 (0%)	0.003**

Data are median [Q1, Q3] for continuous variables and *n* (%) for categorical variables.

*AC* anterior chamber, *CS* ciliary sulcus, *ECD* corneal endothelial cell density, *IOP* intraocular pressure, *SD* standard deviation.

*p*-values: Mann–Whitney U test (continuous) and Fisher’s exact test (categorical). **p < 0.01.

Although posterior tube placement is generally considered protective for the corneal endothelium [2, 3], CS placement was associated with lower baseline-adjusted 1-year ECD in this EXG cohort. Exfoliation material deposits heavily on the ciliary body, zonules, and posterior iris [5], and the proximity of a CS-placed tube to these structures may facilitate the dispersion of exfoliation material into the anterior chamber, potentially contributing to endothelial stress. Given the retrospective design, non-randomised tube selection, small sample size, and baseline imbalances, these results should be considered hypothesis-generating. CS tube placement may not provide the expected endothelial protection in eyes with EXG after BGI, although larger studies are required to confirm this.
